# Robotic-assisted total knee arthroplasty for extra-articular femur deformity correction

**DOI:** 10.1093/jscr/rjad395

**Published:** 2023-07-08

**Authors:** Abdullah A Alturki, Nayf A Alshammari, Abdulrahman L Albassam, Ziad A Aljaafri, Turki S Almugren

**Affiliations:** Department of Orthopedic Surgery, Ministry of the National Guard—Health Affairs, Riyadh, Saudi Arabia; King Saud Bin Abdulaziz University for Health Sciences, Riyadh, Saudi Arabia; Department of Orthopedic Surgery, AlHabib Hospital, Riyadh, Saudi Arabia; College of Medicine, King Saud Bin Abdulaziz University for Health Sciences, Riyadh, Saudi Arabia; College of Medicine, King Saud Bin Abdulaziz University for Health Sciences, Riyadh, Saudi Arabia; Department of Orthopedic Surgery, Ministry of the National Guard—Health Affairs, Riyadh, Saudi Arabia

## Abstract

Femoral shaft fracture is one of the most common injuries encountered. However, improper management can lead to significant long-term complications, of which is malunion. Patients with femoral malunion are at increased risk of developing knee osteoarthritis, and if arthroplasty is indicated, these extra-articular deformities pose a challenge as corrective osteotomy and soft tissue release are also required. In such circumstances, robotic arm-assisted total knee arthroplasty (RATKA) might be an appropriate option. In this case, we present a 66-year-old woman who had previously suffered a femur shaft fracture, which was treated conservatively, and developed a varus malunion and severe knee osteoarthritis, and who was treated with RATKA.

## INTRODUCTION

Femoral shaft fracture is one of the most common injuries. However, improper management can lead to significant long-term complications, which can negatively affect quality of life [1]. One of the most well-known complications is malunion. Patients with malunion, regardless of the cause, are at increased risk of developing knee osteoarthritis, the mechanism of which is most likely distal femur recurvatum, along with varus deformity, distorting the mechanical axis of the knee. The incidence of osteoarthritis in patients with malunion has been reported to be up to 50% [2].

Treatment of patients with femoral malunion and knee osteoarthritis can be problematic. When conservative management fails, surgical correction with total knee arthroplasty (TKA) is preferred. In patients with deformities, conventional TKA is technically challenging, and there is a high risk of implant failure if the deformity is not corrected adequately [3]. Extra-articular deformities can be managed by soft tissue balancing and intra-articular bone resection. However, more severe deformities pose a challenge to surgeons. They require corrective osteotomy and soft tissue release with arthroplasty. In addition, these procedures increase the risk of damaging the medial and lateral collateral ligaments, which may lead to imbalances in the knee joint [4].

Conventional TKA is safe and cost-effective and improves symptoms of patients with knee osteoarthritis. However, patient satisfaction remains an issue, with satisfaction rates ranging between 82% and 89%. This may be attributed to implant failure and the need for revision surgery in patients with preoperative femur fracture and mechanical axis malalignment. In such circumstances, robotic TKA might be an appropriate option. This approach has the advantages of mitigating soft tissue injury and restoring the anatomical mechanical axis [5].

In this case, we present a 66-year-old woman who had previously suffered a femur shaft fracture, which was treated conservatively, and developed a varus malunion and subsequent severe knee osteoarthritis and was treated with a robotic-assisted TKA.

## CASE REPORT

**Figure 1:**
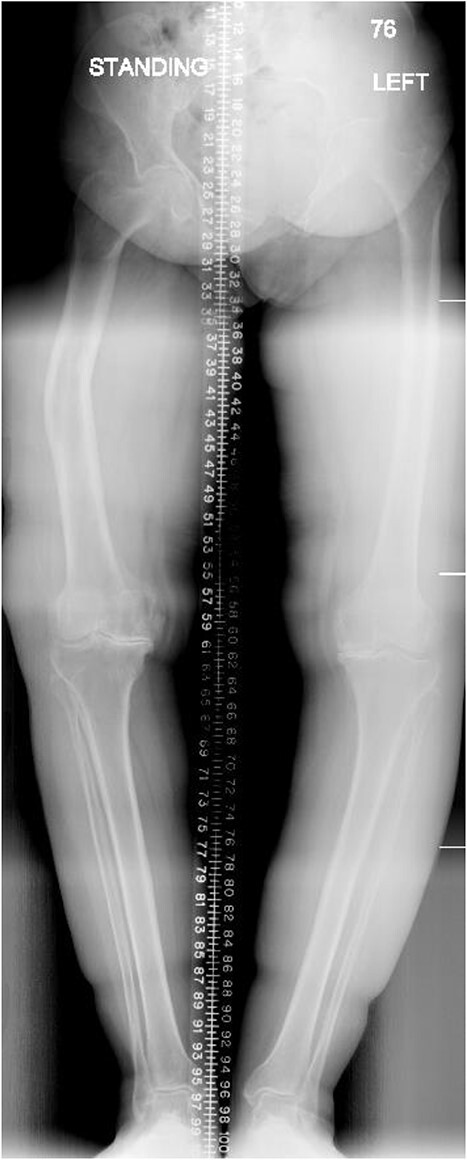
Preoperative anteroposterior standing bilateral lower extremity radiographs displaying long leg alignment and the right femur Varus deformity.

**Figure 2:**
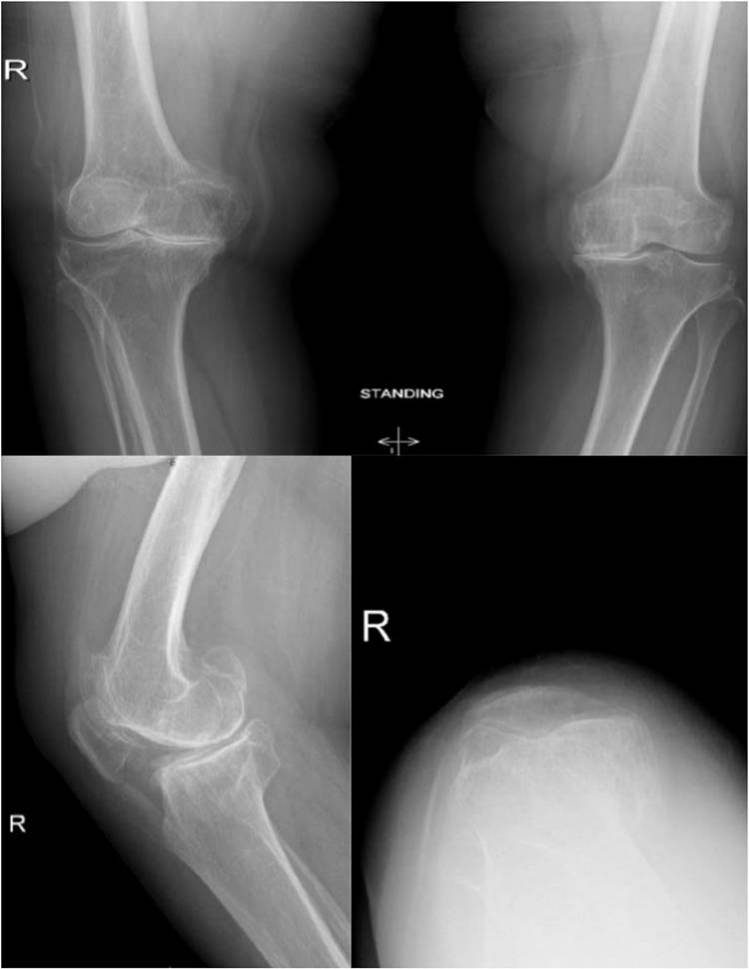
Preoperative anteroposterior, lateral and sunrise radiograph views displaying tri-compartmental osteoarthritis.

A 66-year-old woman with a history of right femoral shaft fracture, which was managed conservatively and resulted in malunion with right varus knee deformity. At the age of 54 years, she was diagnosed with osteoarthritis, and conservative treatment options, including weight reduction, non-steroidal anti-inflammatory medications, physiotherapy and intra-articular steroid injections, had been exhausted. Knee pain continued to worsen over the years, with negligible improvement in her symptoms. On physical examination, there was obvious varus deformity of the right knee, and significant restriction in the range of motion from full extension to 90° flexion. X-rays were requested, which revealed severe tri-compartmental knee osteoarthritis, a 3 cm medial shift of the right lower-limb mechanical axis, and 80° anatomical lateral distal femoral angle (aLDFA) and 96° mechanical lateral distal femoral angle (mLDFA) (Figs 1 and 2). The findings were discussed with the patient, who agreed to proceed with TKA. Given the complexity of this case with the femur deformity, the CORI Smith & Nephew surgical arthroplasty system was recommended as the surgical option that was suited to the patient’s specific bony anatomy and appropriate positioning of the components.

**Figure 3:**
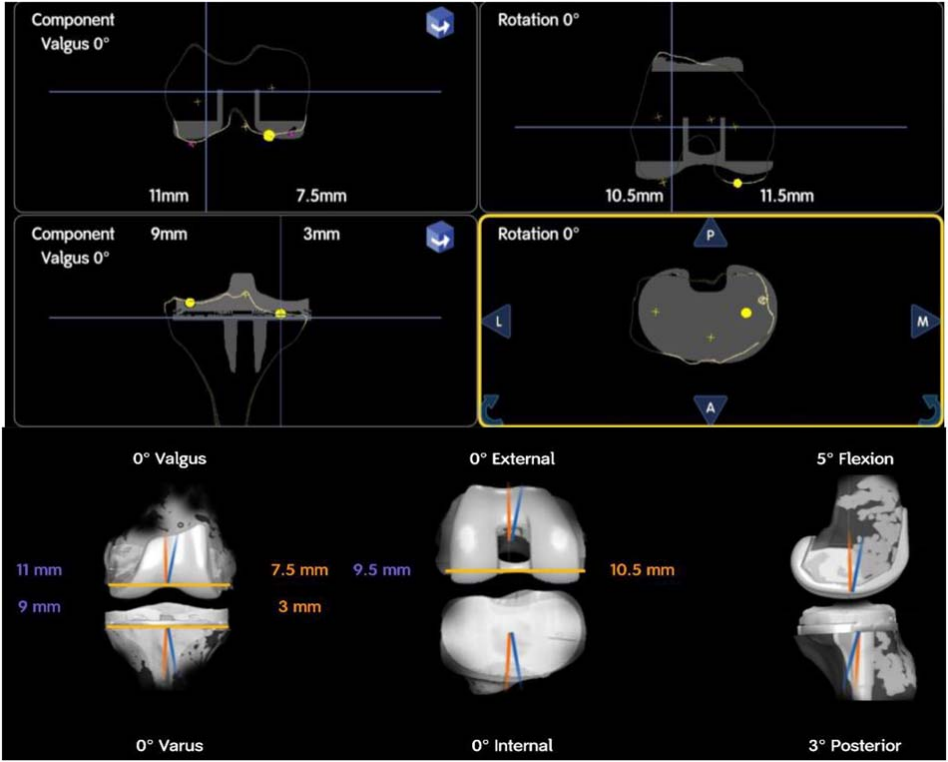
Intra-operative planning of components’ position showing distal femoral cuts: 11 mm medially and 7.5 mm laterally, proximal medial tibial cut is 9 mm and 3 mm laterally.

**Figure 4:**
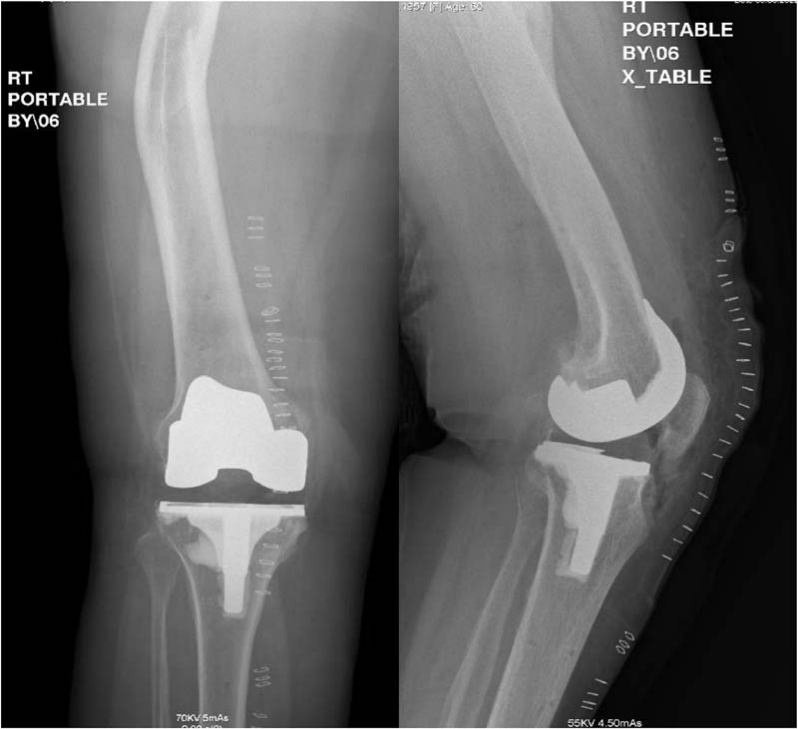
Postoperative anteroposterior and lateral radiograph views of the status of post RATKA.

Spinal regional anesthesia was performed, and 1 g of cephazolin was administered, followed by prepping and draping of the right lower limb in the usual sterile manner. After inflation of an above-knee tourniquet, a right knee midline incision followed by a medial para-patellar arthrotomy was performed. Knee joint exposure with proximal medial tibia release was carried out, followed by application of check points plus two pins in each of the tibia and the femur (navigation receptors). The hip center of rotation was registered, followed by medial and lateral malleolus registration. All large femoral and tibial osteophytes were resected, followed by mapping of articular cartilage in the femur and tibia. After mapping and balancing extension and flexion gaps (Fig. 3), the following sizes were determined: four for the femur and three for the tibia. The distal femur cut was carried out at 90° to the mechanical axis with robotic assistance, utilizing a burr and the remaining femoral cuts were completed with a distal femoral jig and electrical saw. The tibia cut could be achieved with an extramedullary guide, but a burr was utilized for added accuracy regarding the tibial slope and angle of the cut. A trial was performed after completion of the femoral and tibial cuts; it showed good stability in both flexion and extension, with maintained patellar tracking. Copious irrigation with 3 L of normal saline and local anesthesia for the posterior capsule of the knee was performed, followed by cementation and final components placement. Then, the tourniquet was deflated and hemostasis was confirmed, followed by closure in layers and sterile dressing application. Postoperative radiographs showed good positioning of the components (Fig. 4).

The patient stayed in hospital for 2 days after the procedure to ensure good pain control, and mobilization was performed before discharge. She returned for a follow-up visit 3 weeks postoperatively. During that visit, her range of motion was 0°–110° flexion, with stable varus/valgus stress tests and good strength of the quadriceps muscles. A total of 6 months after the procedure, the patient presented to the clinic with no pain and was satisfied. Her final range of motion was 0°–130° flexion.

## DISCUSSION

The positioning of the knee joint is determined by the mechanical axis of the lower extremity, which when not aligned correctly, can cause issues. One of the problems is that the distribution of weight-bearing forces across the joint changes from the medial to the lateral compartment, which can lead to the cartilage deteriorating and the joint having an abnormal range of motion. These factors can cause various knee conditions, such as osteoarthritis, patellofemoral pain syndrome and ligamentous injuries. Thus, it is important to evaluate and address any mechanical axis malalignment to maintain a healthy knee joint and prevent degenerative joint diseases from developing [6]. Sharma *et al.* showed that people with varus misalignment had a higher risk of experiencing progression of medial knee osteoarthritis over a period of 18 months. On the other hand, those with valgus misalignment had an increased risk of developing lateral knee osteoarthritis in the later stages. These findings suggest that the alignment of the knee joint plays a role in the development and progression of knee osteoarthritis [7]. The results of that study suggest that the degree of misalignment at the beginning of the observation period is linked to a more significant decline in physical function over time. This finding highlights the role of body alignment in the risk of developing osteoarthritis and experiencing a decrease in functionality.

Advancements in surgical procedures have revealed the positive effects of robotics in patient care, leading to faster recovery and shorter hospital stays [8]. Incorporating technology in healthcare provides a competitive edge that may result in favorable outcomes. Song *et al.* reported that using the robotic arm-assisted total knee arthroplasty (RATKA) system resulted in greater accuracy in implant positioning, less postoperative bleeding and reduced bone removal compared with traditional jig-based TKA (JTKA) [9]. An intramedullary jig-based technique in deformity cases may result in component mispositioning. We therefore could not depend on the anatomical axis to achieve a perpendicular cut with respect to the mechanical axis of the femur. With robotic software, we were able to plan the implant position in a way that would restore the mechanical axis.

The traditional total knee arthroplasty (JTKA) relies on an anatomical axis blueprint to determine implant positioning. However, patients with osteoarthritis or bone deformity may have misleading joint kinematics due to osteophyte formation and an angulated limb axis. These issues can alter the anatomical axis, making JTKA techniques inadequate. A robotic arm-assisted device considers both the anatomical and the mechanical axes, which is useful for treating altered native joints. Sodhi *et al.* suggested using preoperative CT images to develop a plan for RATKA to evaluate deformities and execute a strategy for balanced and aligned arthroplasty [9]. Preoperative planning and intra-operative feedback from the robotic arm-assisted device help surgeons balance and position the implant while preserving delicate tissues that support intra-articular capsule movement. The implant’s position and joint balance determine the implant’s longevity, which depends on the amount of flexion and extension, corrected angular deformities and tissue preservation [10].

## CONCLUSION

The utilization of RATKA for the management of osteoarthritis with extra-articular femur deformity is appropriate for planning a balanced and aligned arthroplasty. We have presented a case that was managed successfully with no residual complications.
